# Tumorigenicity of Ewing sarcoma is critically dependent on the trithorax proteins MLL1 and menin

**DOI:** 10.18632/oncotarget.13444

**Published:** 2016-11-18

**Authors:** Laurie K. Svoboda, Natashay Bailey, Raelene A. Van Noord, Melanie A. Krook, Ashley Harris, Cassondra Cramer, Brooke Jasman, Rajiv M. Patel, Dafydd Thomas, Dmitry Borkin, Tomasz Cierpicki, Jolanta Grembecka, Elizabeth R. Lawlor

**Affiliations:** ^1^ Department of Pediatrics and Communicable Diseases, University of Michigan Medical School, Ann Arbor, MI 48109, USA; ^2^ Department of Surgery, University of Michigan Medical School, Ann Arbor, MI 48109, USA; ^3^ Department of Pathology, University of Michigan Medical School, Ann Arbor, MI 48109, USA; ^4^ Department of Dermatology, University of Michigan Medical School, Ann Arbor, MI 48109, USA

**Keywords:** Ewing sarcoma, trithorax, MLL, menin, HOX

## Abstract

Developmental transcription programs are epigenetically regulated by the competing actions of polycomb and trithorax (TrxG) protein complexes, which repress and activate genes, respectively. Ewing sarcoma is a developmental tumor that is associated with widespread de-regulation of developmental transcription programs, including HOX programs. Posterior *HOXD* genes are abnormally over-expressed by Ewing sarcoma and *HOXD13*, in particular, contributes to the tumorigenic phenotype. In MLL1 fusion-driven leukemia, aberrant activation of *HOXA* genes is epigenetically mediated by the TrxG complex and *HOXA* gene expression and leukemogenesis are critically dependent on the protein-protein interaction between the TrxG proteins MLL1 and menin. Based on these data, we investigated whether posterior *HOXD* gene activation and Ewing sarcoma tumorigenicity are similarly mediated by and dependent on MLL1 and/or menin. Our findings demonstrate that Ewing sarcomas express high levels of both MLL1 and menin and that continued expression of both proteins is required for maintenance of tumorigenicity. In addition, exposure of Ewing sarcoma cells to MI-503, an inhibitor of the MLL1-menin protein-protein interaction developed for MLL1-fusion driven leukemia, leads to loss of tumorigenicity and down-regulated expression of the posterior *HOXD* gene cluster. Together these data demonstrate an essential role for MLL1 and menin in mediating tumor maintenance and posterior *HOXD* gene activation in Ewing sarcoma. A critical dependency of these tumors on the MLL1-menin interaction presents a potentially novel therapeutic target.

## INTRODUCTION

Normal embryogenesis and tissue maintenance are governed by the coordinated regulation of gene expression in the proper time and space. This process is directed by epigenetic modifications to chromatin that are mediated in part by the large, multi-subunit Polycomb (PcG) and Trithorax (TrxG) complexes [1]. PcG and TrxG proteins, which promote gene repression and activation, respectively, are highly expressed in stem cells and are key epigenetic mediators of both normal development and tumorigenesis [2]. Alterations in the expression, function and composition of these complexes are common features of human malignancy. Deregulation of developmental programs is particularly evident in pediatric cancers, where the normal epigenetic processes governing stem cell self-renewal and differentiation are hijacked, promoting malignant transformation [3]. The best-characterized targets of PcG and TrxG are the *HOX* genes, transcription factors that are critical for normal embryogenesis, development and tissue maintenance [4–6]. Reciprocal epigenetic regulation of *HOX* genes by PcG and TrxG ensures that *HOX* genes are activated in the proper spatiotemporal manner [7]. It is increasingly evident that epigenetic deregulation of *HOX* genes is a hallmark of several cancers, highlighting a critical role for these developmental programs in oncogenesis [8–10].

Ewing sarcoma, an aggressive pediatric bone and soft tissue tumor, is characterized by the presence of EWS/ETS fusion oncogenes, most commonly EWS/FLI1, that arise as a consequence of recurrent chromosomal translocations [11]. The mechanisms by which EWS/ETS fusions induce oncogenic transformation remain to be fully elucidated but increasing evidence suggests that deregulation of epigenetic processes lies at the heart of Ewing sarcoma pathogenesis [12–17]. Although the cellular ontogeny of Ewing sarcoma is unclear, current evidence suggests that it arises via EWS/ETS-dependent malignant transformation of a primitive mesenchymal stem cell of mesoderm or neural crest origin [18, 19]. Consistent with the stem-like phenotype of Ewing sarcomas, the PcG proteins BMI-1 and EZH2 are highly expressed and play well-established roles in tumor pathogenesis [12, 14, 19, 20]. However, we recently reported that despite high levels of BMI-1 and EZH2, a large subset of PcG target genes are paradoxically overexpressed in Ewing sarcoma tumors [15]. Notably, posterior *HOXD* genes, particularly *HOXD13*, are markedly overexpressed by Ewing sarcoma cells and the promoters of these genes are enriched with the TrxG-dependent H3K4me3 histone modification, consistent with a transcriptionally active chromatin state [15, 21].

Post-translational mono-, di-, and trimethylation of H3K4 are mediated by the KMT2 (MLL) family of histone methyltransferases in the context of COMPASS- (Complex of proteins associated with Set1)- and COMPASS-like multi-protein chromatin remodeling complexes [22]. Genome-wide trimethylation of H3K4 (H3K4me3) is largely mediated by *KMT2F* (SETD1A) and *KMT2G* (SETD1B) - containing COMPASS complexes. In contrast, H3K4me3 modifications at the promoters of developmental transcription factors are dependent on TrxG COMPASS-like complexes that contain *KMT2A* (MLL1) and *KMT2B* (MLL4; Mll2 in mice) [22–24]. The MLL family of histone methyltransferases is critical for early embryonic patterning, skeletal development, and hematopoiesis, and these functions are mediated in part by MLL1-dependent activation of *HOX* genes [22, 25–27]. In addition to their critical role in normal development, oncogenic roles for MLL are well-established, especially in leukemia where *MLL1* gene rearrangements and fusion proteins cooperate with the wild-type *MLL1* allele to induce malignant transformation of hematopoietic progenitors via deregulation of *HOXA* and other oncogenic genes [9]. Importantly, the oncogenic function of MLL fusion proteins in leukemia is critically dependent upon their association with the scaffolding protein menin, a partner protein of MLL1 and MLL4 (i.e. *KMT2A* and *KMT2B*) in the TrxG COMPASS-like complexes [28]. Menin is also involved in regulation of *HOX* expression during normal development, hematopoiesis, and in several cancers [10, 23, 26, 29–32]. For the current work, we investigated whether Ewing sarcoma tumorigenicity and *HOX* gene activation are critically dependent on MLL1 and menin. In addition, we have tested if exposure of Ewing sarcoma cells to MI-503, a recently developed inhibitor of the MLL-menin protein-protein interaction [33, 34], impacts on tumorigenicity or posterior *HOXD* gene expression. Our results provide novel insights into tumor pathogenesis and identify a potential therapeutic vulnerability in Ewing sarcoma.

## RESULTS

### MLL1 is highly expressed in Ewing sarcoma tumors and cell lines

**Figure 1:**
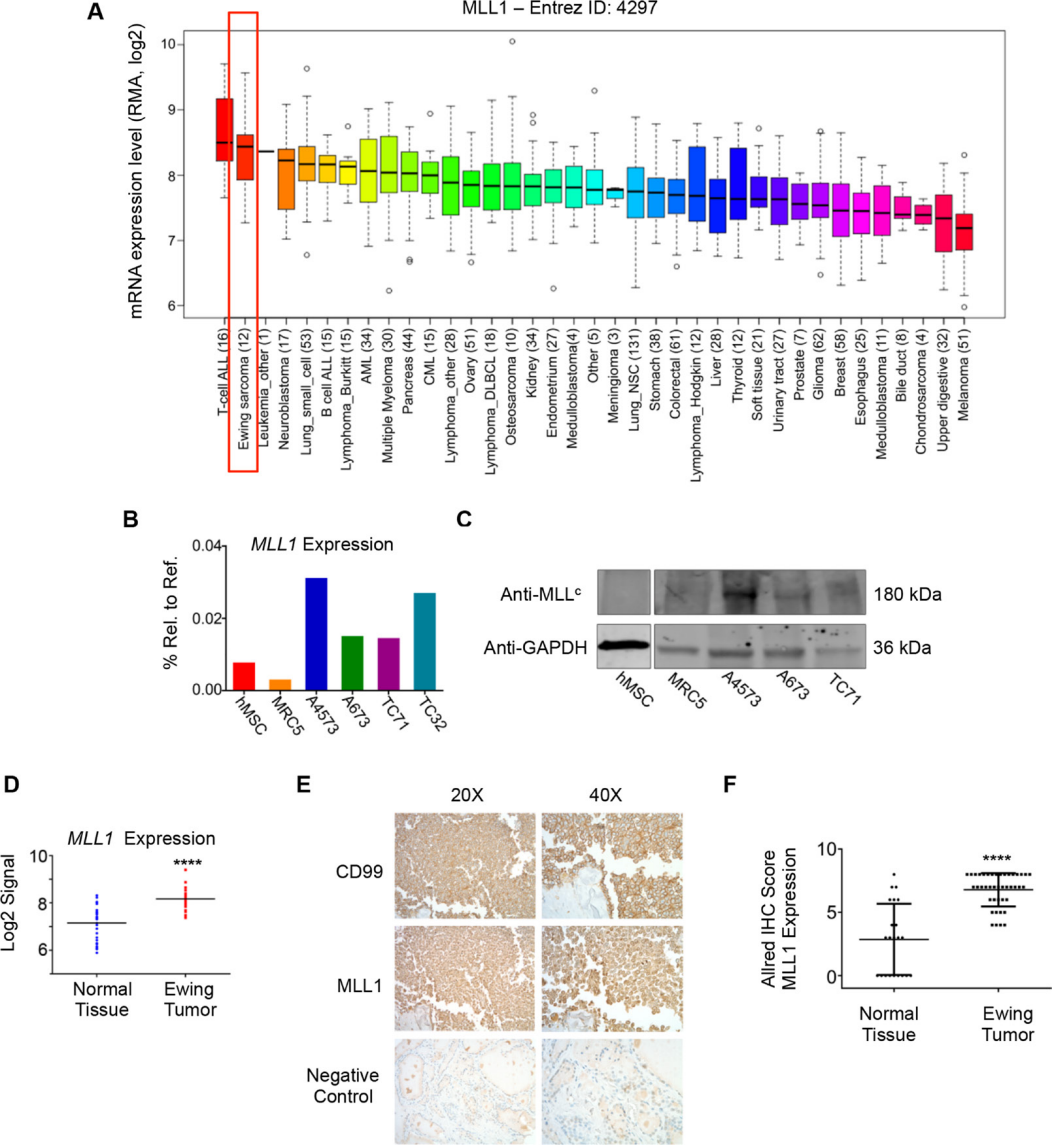
MLL1 is expressed by Ewing sarcoma (**A**) CCLE data depicting MLL1 transcript expression. (**B**) RT-PCR and (**C**) western blot depicting MLL1 transcript and protein expression in Ewing sarcoma cells, MRC5 fibroblasts and adult bone marrow-derived mesenchymal stem cells (hMSC). (**D**) Expression array data for MLL1 in 32 Ewing sarcoma tumors compared to 33 samples from 11 normal adult tissues. ****p < .0001. (**E**) Representative immunohistochemical staining of Ewing sarcoma tumor, showing CD99 (top) and MLL1 (middle). Thyroid (bottom) was negative for MLL1 expression. (**F**) Scoring of MLL staining in 42 Ewing sarcoma tumors and 22 normal tissue samples (liver, bladder, heart, fat, lung, spleen, pancreas, thymus, stomach, salivary gland, thyroid, uterus, kidney, prostate, testes, tonsil, breast, skin). Normal tissues with high MLL1 expression (Allred score of 6 or greater) included heart, spleen, stomach, pancreas, and salivary gland. ****p < .0001.

We and others have shown that the posterior *HOXD* genes, *HOXD9, HOXD10, HOXD11,* and *HOXD13* are overexpressed by Ewing sarcoma [15, 35] and that the promoters of these genes are devoid of the repressive H3K27me3 mark and highly enriched with the MLL-mediated H3K4me3 mark [15, 21]. We therefore hypothesized that MLL may be an important mediator of posterior *HOXD* gene expression and Ewing sarcoma tumorigenicity. To address this question, we first assessed expression of the six MLL family histone methyltransferases in Ewing sarcoma cell lines using the CCLE database [36]. Both *MLL1* (*KMT2A*) and *SETD1B* (*KMT2G*) are highly expressed by Ewing sarcoma cell lines, with expression levels comparable to those of several hematological malignancies known to be driven by MLL rearrangements and/or amplification [9] (Figure 1A and Supplementary Figure S1). Given the well-established role for MLL1 in the deregulation of *HOX* genes in the context of leukemia [9], we focused our studies on MLL1. qRT-PCR analyses of Ewing sarcoma cell lines, bone marrow-derived mesenchymal stem cells (hMSC), putative cells of origin for Ewing sarcoma [18, 19, 37], and non-transformed MRC5 fibroblasts validated the CCLE data, showing robust expression of MLL1 transcript and protein in Ewing sarcoma cells (Figure 1B and 1C). Analysis of published gene expression array data confirmed that *MLL1* is also overexpressed by Ewing sarcoma tumors compared to non-malignant tissues *in vivo* (Figure 1D). Moreover, immunohistochemical analysis of tissue specimens revealed robust expression of MLL1 in 100% of Ewing sarcomas, while expression was detected in only 55% of the control tissues and levels were significantly lower than those expressed by tumors (Figure 1E and 1F). Thus, MLL1 is robustly expressed by Ewing sarcoma cells and tumors *in vitro* and *in vivo*, underscoring a potential role for this proto-oncogene in Ewing sarcoma pathogenesis.

### Loss of MLL1 reduces Ewing sarcoma cell proliferation and tumorigenicity

**Figure 2:**
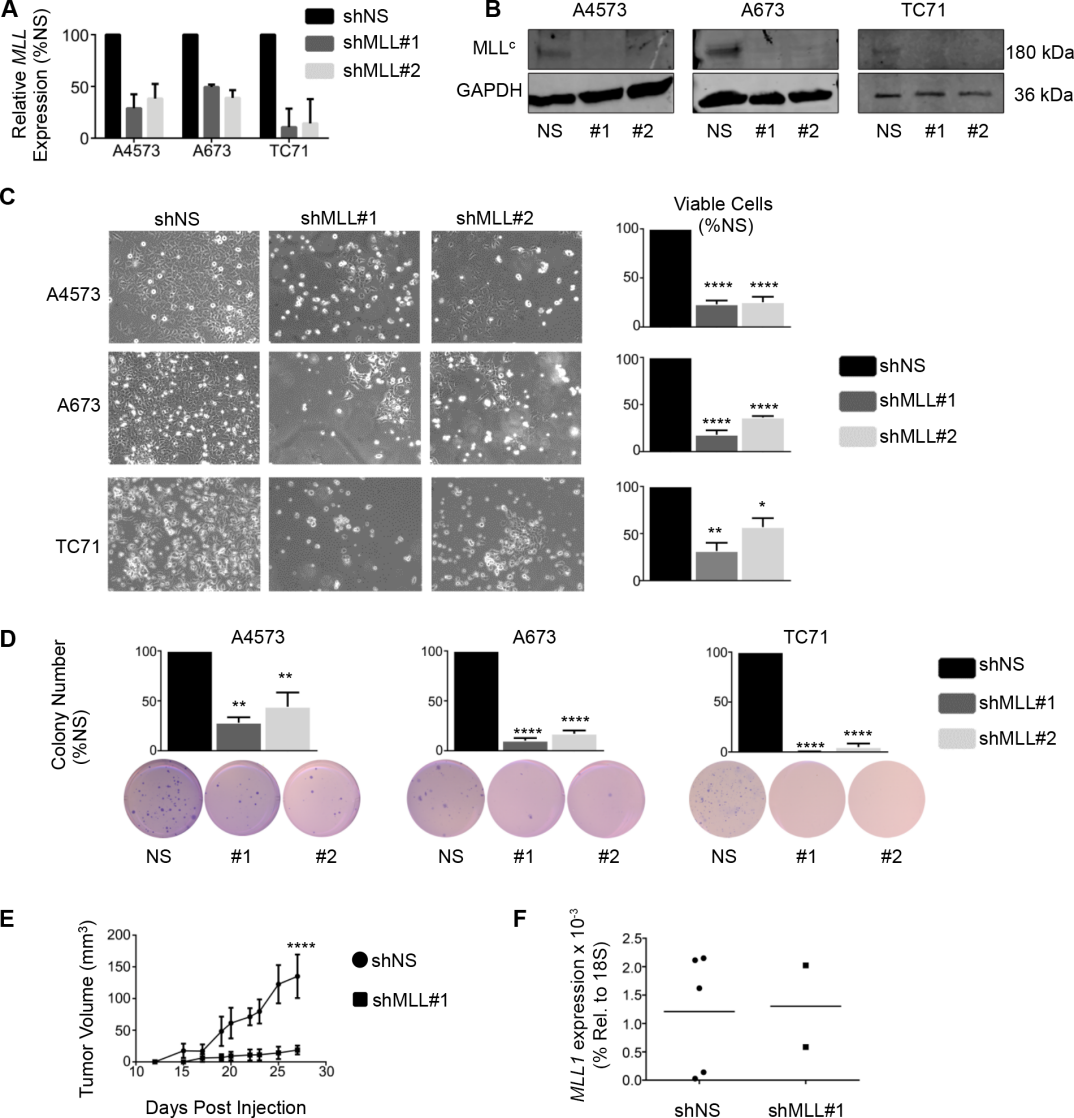
Knockdown of MLL1 in Ewing sarcoma cells reduces proliferation and tumorigenicity qRT-PCR (**A**) and western blot (**B**) showing MLL1 knockdown. (**C**) Brightfield images (left) and histograms (right) show reduced cell expansion after 6 days. (**D**) Histograms (top) and representative images (bottom) showing reduced tumorigenicity with MLL1 knockdown. *N* = 3 experiments. *p < .05, **p = .002, ****p < 0.0001. (**E**) Knockdown of MLL1 significantly reduced tumor growth rate *in vivo*. ****p = .0001. *N* = 5 mice. (**F**) qRT-PCR for *MLL1* shows restoration of *MLL1* expression in tumors derived from shMLL1 cells.

To evaluate a functional role for MLL1 in Ewing sarcoma, we knocked down its expression in 3 Ewing sarcoma cell lines. Knockdown of MLL1 using 2 different lentiviral shRNA constructs (Figure 2A and 2B) dramatically inhibited Ewing sarcoma cell expansion in culture, with evidence of both induction of cell death and reduced cell proliferation (Figure 2C). To investigate a potential role for MLL1 in mediating tumorigenicity, we plated equal numbers of viable cells in soft agar for a period of 2–4 weeks. Knockdown of MLL1 markedly reduced colony formation (Figure 2D), demonstrating that MLL1 promotes Ewing sarcoma tumorigenicity *in vitro*. Finally, we examined whether loss of MLL1 would reduce tumorigenicity *in vivo*. To this end, we injected MLL1 knockdown and non-silencing control cells into contralateral flanks of nude mice. The rate of engraftment, tumor frequency and growth rate of engrafted tumors were all substantially reduced in the context of MLL1 knockdown (Figure 2E). Importantly, qRT-PCR of explanted tumors showed that *MLL1* expression had been restored in the tumors that were derived from shMLL1 cells (Figure 2F). Thus, these data collectively demonstrate that Ewing sarcoma cells are critically dependent upon MLL1 for their continued proliferation, survival and tumorigenicity.

### *HOXD13* is a downstream target of MLL1

**Figure 3:**
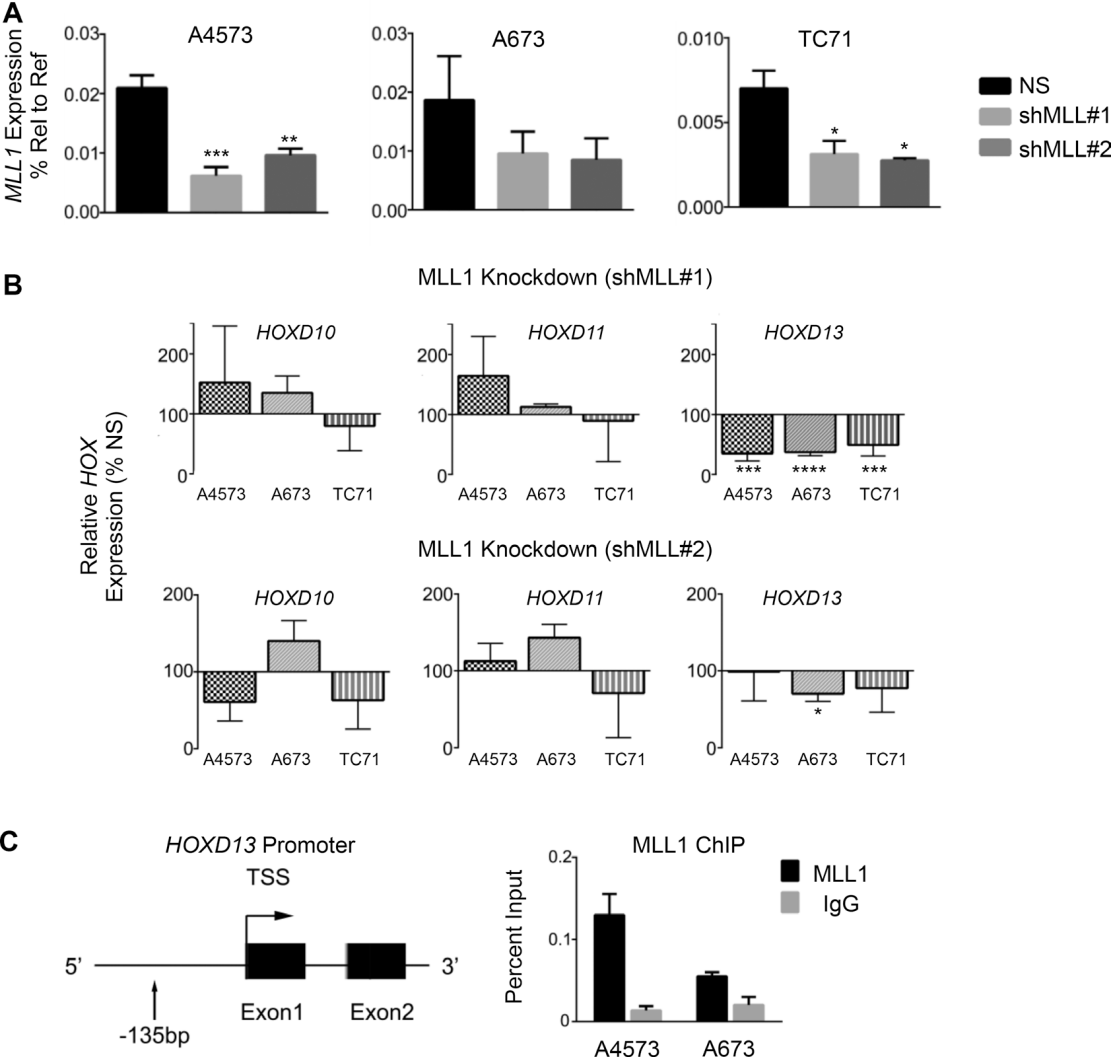
*HOXD13* is a target of MLL1 (**A**) qRT-PCR depicting *MLL1* transcript levels. (**B**) qRT-PCR of posterior *HOXD* gene expression in cells with MLL1 knockdown. Error bars depict mean ± SEM. *p < .05, **p < .01, ***p < .001, ****p < 0.0001. (**C**) Left: Diagram of the *HOXD13* promoter showing the location of primers used for qPCR. Right: MLL1 ChIP at the *HOXD13* promoter. *N* = 2 experiments.

Given the critical role for MLL1 in regulating *HOX* gene expression during normal development and in leukemia [8, 25, 26], we hypothesized that MLL1 contributes to overexpression of posterior *HOXD* genes in Ewing sarcoma. To address this we measured expression of posterior *HOXD* genes 48 hours following shRNA-mediated knockdown of MLL1 (Figure 3A). Studies of altered expression were limited to evaluation of transcript levels due to the lack of specific and authenticated antibodies for *HOXD* protein expression. Although levels of *HOXD10* and *HOXD11* changed in some cells, *HOXD13* was reproducibly downregulated following acute MLL1 knockdown (Figure 3B). Notably, among the posterior *HOXD* genes, *HOXD13* is the most reproducibly and specifically overexpressed by Ewing sarcoma compared to other tumors and non-malignant tissues (Supplementary Figure S2A and Refs [15, 35]). In addition, knockdown of *HOXD13* (Supplementary Figure S2B–2E), and also of *HOXD10* and *HOXD11* [35], results in loss of tumorigenicity, verifying the identity of posterior *HOXD* genes as critical oncogenes in Ewing sarcoma. Directed ChIP-PCR assays confirmed that the presence of H3K4me3 at the *HOXD13* promoter is associated with binding of MLL1 (Figure 3C) [15, 21]. Whether MLL1 or another KMT protein mediates the H3K4me3 modification cannot be ascertained from these data. Nevertheless, given that MLL1 directly activates *HOX* genes in embryonic development, our data lend strong support for the hypothesis that MLL1 also contributes to epigenetic activation of *HOXD13* in Ewing sarcoma. Thus, these data together show that continued high level expression of both MLL1 and HOXD13 are required for maintenance of the oncogenic phenotype of Ewing sarcoma and that *HOXD13* overexpression is associated with H3K4me3 and MLL1 binding at its promoter.

### The scaffolding protein menin has an oncogenic function in Ewing sarcoma

In MLL fusion-driven leukemia, both *HOXA* activation and the oncogenic function of the fusion are critically dependent upon the TrxG scaffolding protein menin [28]. We therefore questioned whether menin might also play an important role in Ewing sarcoma. To test this, we first evaluated menin expression in Ewing sarcoma cell lines and tumors. We again interrogated publically available CCLE gene expression data [36], and found menin (*MEN1*) expression to be robust in Ewing sarcoma (Supplementary Figure S3A). Furthermore, we found that menin transcript and protein are highly expressed by Ewing sarcoma cell lines compared to non-transformed cells (Supplementary Figure S3B and S3C). Likewise, Affymetrix expression array data showed significantly higher *MEN1* expression in Ewing sarcoma tumors compared to non-transformed tissues (Supplementary Figure S3D).

**Figure 4:**
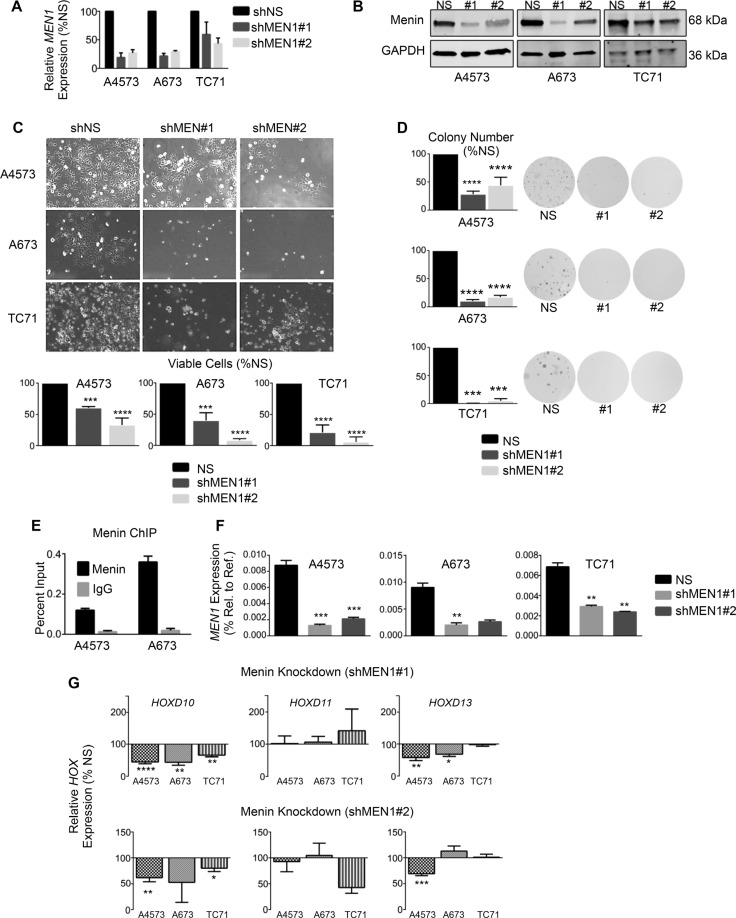
Menin has an oncogenic function in Ewing sarcoma qRT-PCR (**A**) and western blot (**B**) showing menin knockdown. (**C**) Brightfield images (top) and histograms (bottom) show reduced cell growth with menin knockdown. (**D**) Histograms (left) and representative images (right) of soft agar assays. (**E**) Menin ChIP at the *HOXD13* promoter. *N* = 2 experiments. (**F**–**G**) qRT-PCR depicting expression of menin (F) and posterior *HOXD* genes (G). *p < .05, **p < .01, ***p < .001, ****p < 0.0001. *N* = 3 experiments.

To determine whether menin has a functional role in Ewing sarcoma pathogenesis, we knocked down menin transcript and protein expression in Ewing sarcoma cell lines. Knockdown of menin (Figure 4A and 4B) phenocopied MLL1 knockdown, inducing a pronounced reduction in cell expansion in all cell lines (Figure 4C). Colony formation in soft agar was similarly reduced with menin knockdown (Figure 4D), underscoring its importance in promoting tumorigenicity *in vitro*. Notably, knockdown of menin was difficult to achieve, and a substantial amount of protein remained after transduction with lentiviral shRNA (Figure 4B). In spite of this, even partial knockdown of menin was sufficient to yield a robust and reproducible phenotype, underscoring the exceptional dependence of Ewing sarcoma cells on menin for their proliferation, survival and tumorigenicity. We next examined whether menin, like MLL1, is critical for the regulation of *HOXD13*. ChIP studies confirmed binding of menin at the *HOXD13* promoter in Ewing sarcoma cells (Figure 4E). Knockdown of menin caused a modest reduction in *HOXD13* expression in 2 of 3 cell lines, as well as downregulation of *HOXD10* (Figure 4F and 4G). Taken together, these data collectively demonstrate that, like MLL1, menin contributes to the oncogenic Ewing sarcoma phenotype and to maintenance of posterior *HOXD* gene expression.

### Pharmacologic inhibition of the menin-MLL protein-protein interaction inhibits Ewing sarcoma growth and tumorigenicity

**Figure 5:**
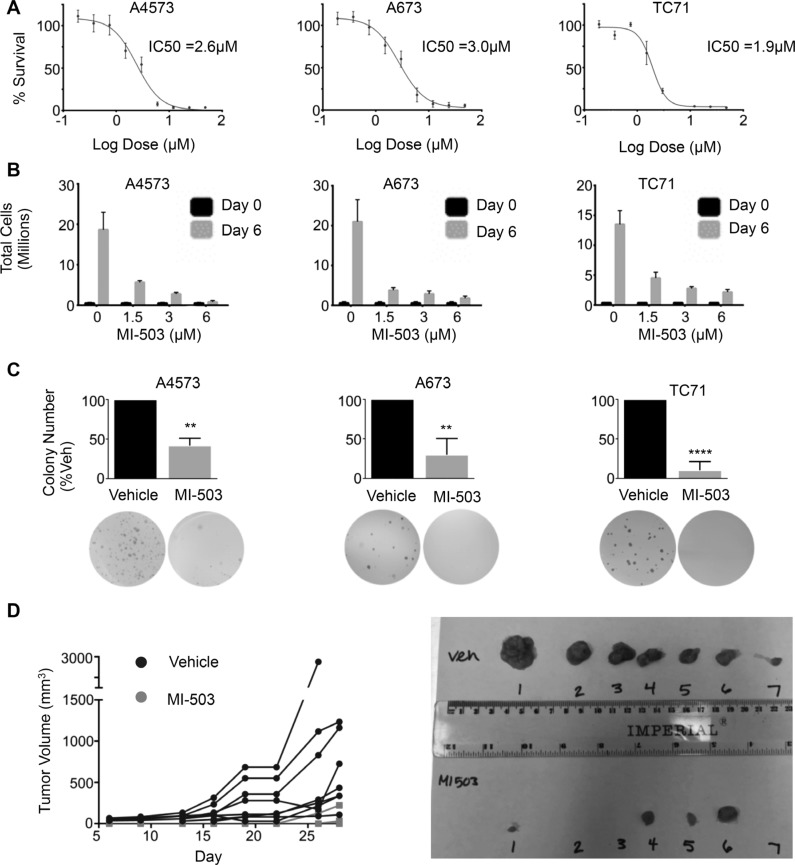
MI-503 inhibits Ewing sarcoma cell expansion and tumorigenicity (**A**) IC_50_ curves and (**B**) trypan blue assay in MI-503 treated cells. (**C**) Histograms (top) and representative images (bottom) of soft agar assays in cells pre-treated with MI-503 or vehicle. *N* = 3 experiments. **p = .002, ****p < 0.0001. (**D**) Left: Spider plot depicting growth rates of individual tumors pre-treated with vehicle (Black Circle) or 6 μM MI-503 (Grey Square). *N* = 8 mice. Right: Image of tumors extracted on day 28.

Having established that MLL1 and menin have critical oncogenic functions in Ewing sarcoma, we hypothesized that they may represent a novel therapeutic target. Small molecule inhibitors of the MLL-menin protein-protein interaction have recently been developed and have been shown to inhibit both oncogenic *HOXA* gene expression as well as tumor propagation in MLL fusion-driven leukemias [33]. Importantly, the inhibitors have also shown preclinical efficacy in tumors that do not harbor MLL fusions, including prostate cancer and glioma [38, 39]. We therefore treated Ewing sarcoma cell lines with the MLL-menin inhibitor, MI-503 [33]. MI-503 treatment resulted in robust and reproducible inhibition of Ewing sarcoma viability (Figure 5A). Notably, the IC_50_ values for the compound were similar to those observed in castration resistant prostate cancer cells, where MLL and menin have been shown to possess tumorigenic functions [39]. In addition, although Ewing sarcoma cells continued to proliferate in the presence of the inhibitor, their rate of proliferation was reduced in a dose-dependent manner (Figure 5B). Moreover, higher doses of the inhibitor induced cell death (Supplementary Figure S4A). In contrast, treatment with an inactive control compound, MI-NC, a very weak inhibitor of the MLL-menin protein-protein interaction [34], at the same doses had no effect on proliferation or cell viability (Supplementary Figure S4B). In addition, control adipose-derived human mesenchymal stem cells were more resistant to MI-503 (IC_50_ = 6.3 μM, Supplementary Figure S4C), suggesting that Ewing sarcoma cells are differentially sensitive to inhibition of the MLL-menin protein-protein interaction. To test the effect of MI-503 on Ewing sarcoma tumorigenicity *in vitro*, we pre-treated Ewing sarcoma cells with MI-503 for 6 days and then plated equal numbers of viable cells in soft agar for 2–4 weeks in the absence of compound. MI-503 pre-treatment led to a significant reduction in colony formation in soft agar, demonstrating that MI-503 reduces Ewing sarcoma cell tumorigenicity *in vitro* (Figure 5C). Next, we sought to determine whether MI-503 would block tumorigenicity *in vivo*. To address this question, we injected equal numbers of viable A4573 cells pre-treated with MI-503 or DMSO control into contralateral flanks of nude mice. Cells pre-treated with vehicle formed tumors with a median time to engraftment of 7.5 days. In contrast, pre-treatment of cells with MI-503 delayed the median time to tumor engraftment to 28 days (Figure 5D). Taken together, these data demonstrate that Ewing sarcoma cells are critically dependent on the MLL-menin protein-protein interaction for their tumorigenicity.

### MI-503 down-regulates posterior *HOXD* genes and MLL1 and menin

**Figure 6:**
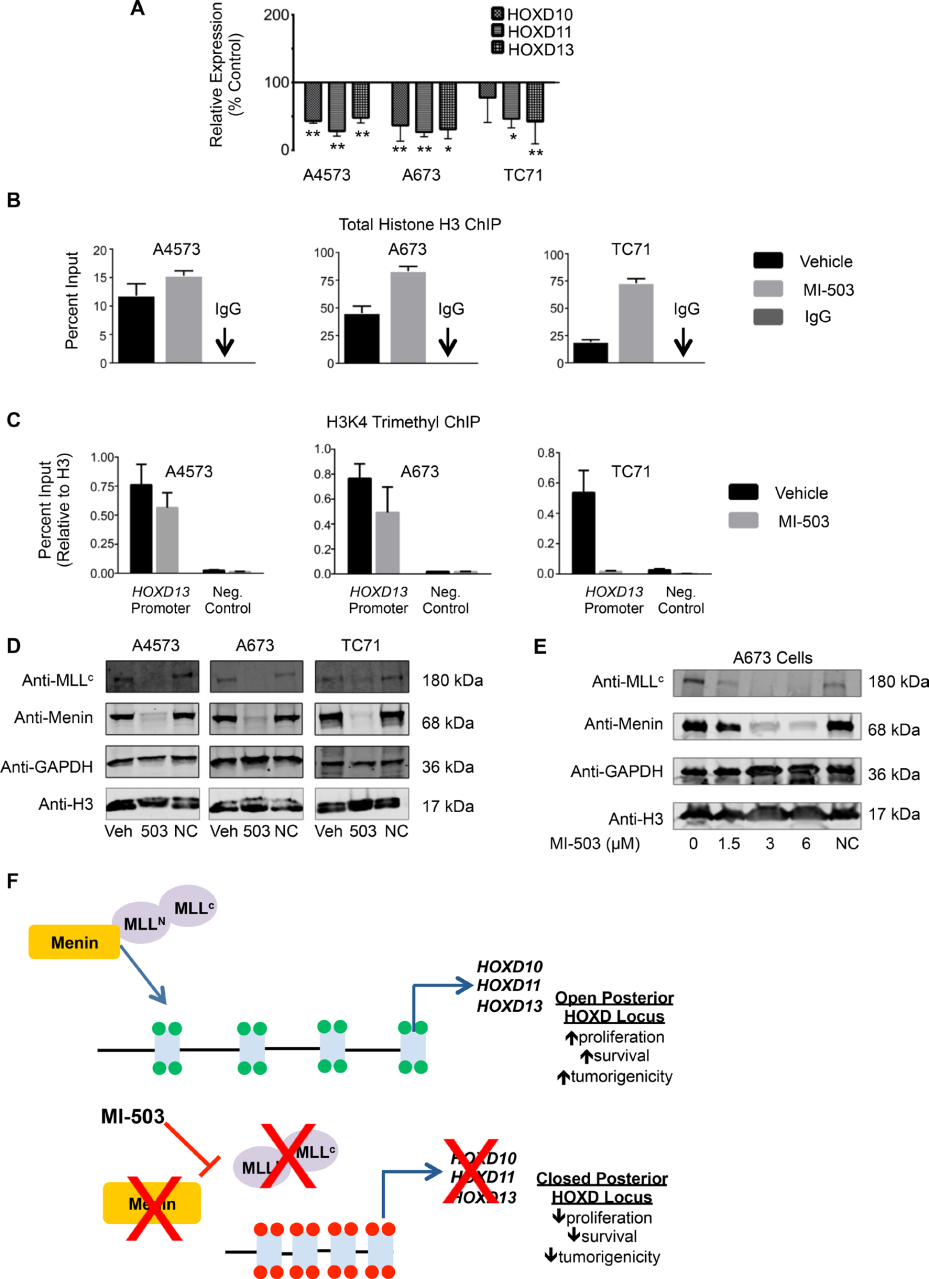
MI-503 treatment of Ewing sarcoma leads to loss of posterior *HOXD* gene and MLL and menin protein expression (**A**) qRT-PCR data depicting reduced *HOXD10*, *HOXD11*, and *HOXD13* expression with MI-503 treatment. *p < .05, **p = .003. (**B**) Increased histone H3 at the *HOXD13* promoter after MI-503 treatment. (**C**) Reduced H3K4me3 at the *HOXD13* promoter with MI-503 treatment. Control is a gene desert region telomeric to the posterior HOXD cluster. Error bars in all panels depict mean ± SEM. (**D**). Western blot depicting MLL1 and menin protein expression after 6 day treatment with MI-503 or MI-NC. (**E**) Western blot showing dose-dependent down-regulation of MLL1 and menin expression with MI-503 treatment. (**F**) Model of epigenetic activation of posterior *HOXD* gene cluster in Ewing sarcoma that is disrupted by exposure of cells to MLL-menin interaction inhibitor.

We next assessed whether, like MLL-fusion driven leukemia, disruption of the MLL-menin interaction inhibits expression of oncogenic HOX programs in Ewing sarcoma cells. As shown, MI-503 treatment was accompanied by robust and reproducible down-regulation of the posterior *HOXD* gene cluster as evidenced by reduced expression of *HOXD10, HOXD11* and *HOXD13* following compound exposure (Figure 6A). ChIP studies revealed that MI-503 treatment caused an increase in total histone H3 levels at the *HOXD13* promoter, consistent with chromatin compaction (Figure 6B). Moreover, this increase in total H3 levels was accompanied by a reduction in H3K4me3 enrichment, consistent with epigenetic repression and decreased transcriptional activity (Figure 6C). Importantly, MI-503 treatment had no effect on global histone H3 levels (Figure 6D and 6E). Notably, our studies also revealed the entirely unexpected finding that MI-503 induced a profound loss of both MLL1 and menin protein expression (Figure 6D). Loss of MLL1 and menin was dose-dependent and occurred in as little as 24 hours (Figure 6E and Supplementary Figure S5A). MI-503 treatment had no consistent effect on *MLL1* and *MEN1* transcript levels (Supplementary Figure S5B and S5C), suggesting that the mechanism for regulation is posttranscriptional. This finding of down-regulated MLL and menin protein expression in response to MI-503 appears to be unique to Ewing sarcoma since, in both leukemia and prostate cancer cells, MI-503 blocks the MLL-menin protein-protein interaction, without affecting their overall expression [33, 39]. Collectively, these data demonstrate that interrupting the MLL-menin protein-protein interaction in Ewing sarcoma results in loss of expression of MLL1, menin and posterior *HOXD* genes, thereby inhibiting a critical tumor-sustaining oncogenic program (Figure 6F).

## DISCUSSION

Increasing evidence suggests that Ewing sarcoma is driven by altered epigenetic regulation of transcriptional programs downstream of the tumor-initiating EWS/ETS fusions [12–16]. In this work we have uncovered a previously unappreciated and essential oncogenic role for the TrxG complex in Ewing sarcoma pathogenesis. In particular, our findings demonstrate that the TrxG proteins MLL1 and menin independently contribute to maintenance of Ewing sarcoma cell survival and tumorigenicity. In addition, pharmacologic studies with a small molecule inhibitor of the MLL-menin protein-protein interaction show that this interaction is essential for both maintenance of tumorigenicity as well as maintenance of expression of MLL1, menin and the oncogenic posterior *HOXD* program. Thus, these studies highlight a potential therapeutic opportunity for Ewing sarcoma that could exploit ongoing TrxG-targeted strategies that are in preclinical and early clinical development for the treatment of MLL-driven leukemia [40].

Our findings add to a growing body of evidence highlighting a tumorigenic role for MLL and menin in solid tumors. Tumor suppressive roles for both MLL and menin have been extensively characterized [22, 41–43]. It is increasingly evident, however, that MLL and menin play a central oncogenic role in several cancers, in part through deregulation of pathways that control embryonic development [8, 10, 28, 44, 45]. During normal development, TrxG and PcG proteins have antagonistic roles in the regulation of developmental gene expression, particularly *HOX* genes [7, 25]. We recently reported that the posterior *HOXD* locus is highly enriched with the MLL-mediated H3K4me3 mark and is nearly devoid of the PcG-mediated H3K27me3 mark [15]. Consistent with a gain of TrxG function in Ewing sarcoma, our data demonstrate that MLL1 and menin contribute to the persistent overexpression of posterior *HOXD* genes in Ewing sarcoma and that this overexpression is highly dependent on the MLL1-menin protein-protein interaction. Thus, our results suggest that, similar to MLL fusion-driven leukemias [46] and glioblastoma [8], aberrant TrxG-mediated activation of developmental HOX programs plays a central role Ewing sarcoma pathogenesis.

*HOXD10*, *HOXD11* and *HOXD13* play essential roles in maintaining the oncogenic phenotype of Ewing sarcoma proliferation, invasion and metastasis (current study and Ref. [35]). The current work sheds further light on the molecular mechanisms that contribute to posterior *HOXD* gene deregulation in this disease. Specifically, our findings strongly implicate MLL1 and menin in mediating epigenetic activation of the *HOXD* locus. It is interesting to note, however, that despite marked inhibition of cell proliferation and tumorigenicity, *HOXD10, HOXD11,* and *HOXD13* gene expression were only modestly reduced when MLL1 or menin were knocked down individually. In contrast, MI-503 treatment, which abolishes expression of both MLL1 and menin, caused marked down-regulation of the cluster. Thus, it is plausible that MLL1 and menin may each be necessary, but not sufficient, for full activation of the posterior *HOXD* locus and that disruption of the MLL1-menin interaction effectively abrogates TrxG complex-dependent epigenetic activation of posterior *HOXD* gene expression. It is important to note, however, that in addition to partnering with MLL1, menin has numerous other context-specific binding partners that are responsible for mediating its highly diverse and cell type-specific functions [47]. Moreover, MI-503 targets a binding site on menin that is critical not only for its interaction with MLL1 (*KMT2A*) but also its interactions with MLL4 (*KMT2B*) and with JUND [33, 47]. Thus, it is possible that the impact of MI-503 on tumorigenicity is secondary to loss of menin interactions with other partner proteins and/or to loss of menin expression itself rather than to specific inhibition of the MLL1-menin interaction. Our observation that Ewing cells rapidly select against menin knockdown supports this possibility, and we are now actively investigating the additional molecular targets of menin in Ewing sarcoma. Identification of the molecular targets of menin, both alone and in the context of MLL1-containing TrxG complexes, will yield important insight into the independent and overlapping roles of menin and MLL1 in tumor pathogenesis.

During embryonic development the posterior *HOXD* genes are coordinately activated in discrete spatiotemporal contexts by several enhancer-like sequences that interact in three-dimensional space [48]. We previously reported that expression of EWS/FLI1 in differentiating stem cells coordinately induces expression of *HOXD10*, *HOXD11* and *HOXD13* along with neighboring transcripts that are also under the control of the same enhancer elements [15]. In the current work, we have shown that pharmacologic inhibition of the MLL-menin interaction leads to concomitant loss of expression of all posterior *HOXD* genes, supporting the potential contribution of developmental enhancers to gene regulation. Significantly, recent epigenomic profiling studies showed that EWS/FLI1 binds to gene enhancers and that binding at these sites overlaps with binding of WDR5, another core component of the TrxG complex [17]. Thus, we speculate that TrxG-dependent activation of the posterior *HOXD* locus might involve cooperation between EWS/FLI1, MLL1 and/or menin, and WDR5 and that these proteins together orchestrate enhancer activation and persistent overexpression of the entire posterior *HOXD* cluster in aggregate. Whether menin and/or MLL1 directly or indirectly contribute to enhancer activation remains to be determined and studies are currently ongoing to test this hypothesis.

In summary, we have discovered that Ewing sarcoma cells are exceptionally dependent upon MLL1 and menin for their oncogenic phenotype. In particular, we have demonstrated that protein-protein interaction between MLL1 and menin is required for continued high-level expression of both MLL1 and menin proteins and of oncogenic posterior *HOXD* genes. This critical dependence of Ewing sarcoma on MLL-menin-mediated activation of a developmental HOX program is highly reminiscent of MLL-fusion positive leukemias and presents a novel opportunity for therapeutic intervention.

## MATERIALS AND METHODS

### Ethics statement

Investigation has been conducted in accordance with the ethical standards and according to the Declaration of Helsinki and according to national and international guidelines and has been approved by the authors' institutional review board.

### Cell lines and stem cells

Ewing sarcoma cell lines were kindly provided by Dr. Timothy Triche at Children's Hospital Los Angeles (CHLA) 2004; Dr. Heinrich Kovar (CCRI, St. Anna Kinderkrebsforschung, Vienna, Austria) 2010; and the Children's Oncology Group (COG) cell bank (cogcell.org), 2012. Identities were confirmed by short tandem repeat (STR) profiling. Cell lines were cultured according to COG- or ATCC-recommended protocols. Human bone marrow-derived mesenchymal stem cells (hMSC) were kindly provided by Dr. Paul Krebsbach (University of Michigan; 2013) and cultured as outlined previously [18]. All cell lines were routinely tested for mycoplasma contamination using the e-Myco^TM^ plus Mycoplasma PCR Detection Kit (Bulldog Bio, Portsmouth, NH).

### Tissue microarray and immunohistochemistry

The University of Michigan Institutional Review Board provided a waiver of informed consent (HUM00067293) to obtain formalin-fixed, paraffin-embedded tissue blocks of 61 Ewing Sarcoma cases (54 primary tumors and 7 metastatic) from the Department of Pathology, University of Michigan Medical Center. Tissue microarray and immunohistochemistry were performed according to previous protocols [49] (See Supplementary Methods). Expression of MLL was scored semi-quantitatively using the Allred schema [50]: Intensity (0–3) and proportion of immune-positive tumor cells (0, none; 1, < 1%; 2, 1–10%; 3, 10–33%; 4, 33–66% and 5, > 66%) were added to produce an Allred IHC score.

### Gene expression data

Publically available gene expression data for Ewing sarcoma cell lines were obtained from the Cancer Cell Line Encyclopedia (CCLE) [36]. Expression array data for primary Ewing sarcoma tumors (GSE68776) and normal adult tissues (www.netaffx.com) were obtained from previously published datasets [15].

### Gene knockdown studies

Lentivirus production and lentiviral gene transfer were conducted as previously described [18] (see Supplementary Table S1).

### *In vitro* proliferation and soft agar assays

Cell proliferation was assessed using the real time xCELLigence impedance- based system (ACEA Biosciences, San Diego, CA) MTS assay or trypan blue exclusion. Soft agar, trypan blue and MTS assays were performed as outlined previously [12, 51]. See Supplementary Methods.

### Chemical synthesis of menin-MLL inhibitors and treatments

MI-503 and MI-NC were prepared using the synthetic procedures reported previously [34]. Cells were treated for 6 days with 3μM MI-503 or DMSO vehicle for proliferation and ChIP assays. Fresh compound was added on day 3 of treatment.

### *In vivo* tumor xenograft studies

All animal studies were performed according to approved protocols per review by the UM Institutional Animal Care and Use Committee (PRO#00006703). For MLL1 knockdown, A4573 cells were transduced with shNS or shMLL#1 lentiviral constructs and used for xenograft studies 48 hours post-transduction. For MI-503 studies, A4573 cells were pre-treated with 6μM MI-503 or DMSO control for 6 days. 250,000 viable cells were then suspended in 50% (v:v) PBS:Matrigel® (Corning, Tewksbury, MA) and injected into contralateral flanks of female NCR nude mice, 8–10 weeks of age (Taconic Farm, Inc., shNS or vehicle-treated cells on left flank, shMLL#1 or MI-503 pre-treated cells on right flank). For HOXD13 knockdown xenograft studies, 500,000 A673 cells harboring doxycycline-inducible HOXD13 knockdown (shHOXD13#1) or non-silencing control were suspended in 50% (v:v) PBS:Matrigel^®^ and injected sub-cutaneously into female NOD-SCID mice, 8–10 weeks of age (Charles River Breeding Labs). Mice were administered water with 2 mg/ml doxycycline and 2% sucrose daily. For all xenograft studies, tumor growth was monitored 2–3 times weekly using digital calipers, and “engraftment” was defined as the presence of a palpable tumor.

### Chromatin immunoprecipitation

Chromatin immunoprecipitation (ChIP) to assess binding/enrichment of MLL, menin, H3K4me3 and total histone H3 were performed using the Zymo-Spin ChIP kit (Zymo Research Corp, Irvine, CA) and quantitative PCR was performed as outlined previously [15]. The following antibodies were used: Anti-menin (Bethyl A300–105A), 4 μg; anti-MLL (Millipore 05–765), 10μg, anti-H3K4me3 (Invitrogen 49–1005), 2 μg, anti-histone H3 (Cell Signaling Technology 2650), 15 μg. Non-immune rabbit or mouse IgG were used as negative controls. Primer sequences for the *HOXD13* promoter and negative control region are detailed in Supplementary Table S2.

### Western blot

Western blot was performed according to established protocols with the following primary antibodies: Anti-menin 1:1000 (Bethyl A300–105A), Anti-MLL^c^ 1:500 (Millipore 05–765), Anti-histone H3 1:1000 (Cell Signaling Technology 2650) and Anti-GAPDH 1:1000 (Cell Signaling 14C10). Membranes were probed with the appropriate secondary antibodies from LiCor Biotechnology (Lincoln, NE) at 1:10,000 dilution and imaged with a LiCor Odyssey infrared imager.

### Statistics

All data are presented as the mean ± SEM, and statistical significance was determined using unpaired, 2-tailed Student's *t*-tests. For tumor growth studies, statistical analysis was conducted by comparing the data at the final time point of the growth curves. Statistical analysis of time to tumor engraftment was conducted using log-rank (Mantel-Cox) test. *P* values of less than 0.05 were considered significant. Analyses were conducted using Graphpad Prism software.

## SUPPLEMENTARY MATERIALS


