# Supplementary material for: Tumorigenicity of Ewing sarcoma is critically dependent on the trithorax proteins MLL1 and menin
Source: Oncotarget. 2016 Nov 18;8(1):458–71. doi: 10.18632/oncotarget.13444 (PMC5352134; doi:10.18632/oncotarget.13444)
Supplement: Supplementary file 1 [file oncotarget-08-458-s001.pdf]

Tumorigenicity of Ewing sarcoma is critically dependent on the trithorax proteins MLL1 and menin

Supplementary Materials

Quantitative RT-PCR

RNA was extracted using the Zymo Quick-RNA Miniprep kit (Zymo Research Corp, Irvine, CA), and cDNA was generated from RNA using the iScript kit (Bio-Rad, Hercules, CA, USA). Quantitative RT-PCR experiments were conducted using Taqman gene expression assays (Life Technologies), with the following probes: *KMT2A* (Hs00610538_m1)*, MEN1* (Hs00610538_m1) *HOXD13* (Hs00968515_m1), *HOXD10* (Hs00157974_m1)*, HOXD11* (Hs00360798_m1), *18S* (Hs03003631_g1), *B2M* (Hs00984230_m1).

Gene knockdown studies

For constitutive knockdown of MLL1 (*KMT2A*) and menin (*MEN1*) expression, shRNA oligonucleotides in the pLKO.1-puro lentiviral vector were purchased from Sigma (see Supplementary Table S1). For MLL1 knockdown, cells were transduced with lentiviral shRNA constructs targeting the coding sequence of the *KMT2A* gene. Functional assays were conducted 48 hours after lentiviral transduction. For menin knockdown, cells were transduced with lentiviral shRNA constructs targeting the 3′UTR (shMEN#1) or the coding sequence (shMEN#2) of the *MEN1* gene, or non-silencing control. Cells received one transduction, followed by a second after 48 hours. Cells were used for functional assays after 4–5 days. Knockdown of *HOXD13* was achieved using the lentiviral doxycycline-inducible pTRIPZ shRNA system (GE Dharmacon, Supplementary Table S1). After lentiviral transduction, cells were selected in 1.5 µg/mL puromycin for 48 hrs, and induced with 1 µg/mL doxycycline for 72 hrs. *HOXD13, MEN1* and *MLL1* (*KMT2A)* knockdown were validated by Taqman quantitative RT-PCR.

Chromatin immunoprecipitation

PCR of ChIP samples was performed using shown in primers Supplementary Table S2.

Proliferation assays

For xCELLigence proliferation assays, cells were pre-treated with 1µg/mL doxycycline for 48 hours to induce knockdown of *HOXD13*. 1×10^4 cells were then seeded in E-plates with 200mL growth medium and permitted to grow up to 72 hours. Cellular impedance was measured every 30 minutes. Data were normalized to the 12-hour time point, at which all cells had adhered to the plate.

Tissue microarray and immunohistochemical staining

To construct tissue microarray, each case was represented by single 1 mm diameter cores, obtained from the most representative, non-necrotic area of the tumor. In addition, 26 Ewing Sarcoma cell lines were also arrayed along with control tissue and orientation cores. Immunohistochemical staining was performed on the DAKO Autostainer (DAKO, Carpinteria, CA) using DAKO Envision+ and diaminobenzadine (DAB) as the chromogen. De-paraffinized TMA sections were labeled with MLL-2829 (rabbit polyclonal antibody, 1:500, a kind gift of Dr. Yali Dou, Department of Pathology, University of Michigan) for 30 minutes at ambient temperature. Microwave citric acid epitope retrieval was used prior to staining. Appropriate negative (no primary antibody) and positive controls were stained in parallel with each set of tumors studied.

Supplementary Table S1: shRNA sequences

Gene	Oligo ID	Sequence
shMLL1#1	TRCN0000005954 (Sigma)	CCGGGCACTGTTAAACATTCCACTT CTCGAGAAGTGGAATGTTTAACAGTGCTTTTT
shMLL1#2	TRCN0000005956 (Sigma)	CCGGCGCCTAAAGCAGCTCTCATTT CTCGAGAAATGAGAGCTGCTTTAGGCGTTTTT
shMEN1#1	TRCN0000338276 (Sigma)	CCGGCCTCACGCTGGGTCCTAATTACT CGAGTAATTAGGACCCAGCGTGAGGTTTTTG
shMEN1#2	TRCN0000338331 (Sigma)	CCGGCTGTACCTGAAAGGATCATACCTC GAGGTATGATCCTTTCAGGTACAGTTTTTG
shHOXD13#1	V3THS_321416 (GE Dharmacon)	TCTTGTCCTTCACTCTTCG
shHOXD13#2	V2THS_93475 (GE Dharmacon)	TATTCAAGTCTTTCCAAAG

Supplementary Table S2: ChIP qPCR primer sequences

Primer set	Fwd	Rev
HOXD13	CCCCTCCCAACCTGAACTTC	CCTCTAGCCCTCTCTCCCTC
HOXD cluster negative control	GTTTTGAGCTGCCCTATGGA	TGCAGCTGTCATAGCGAGAG

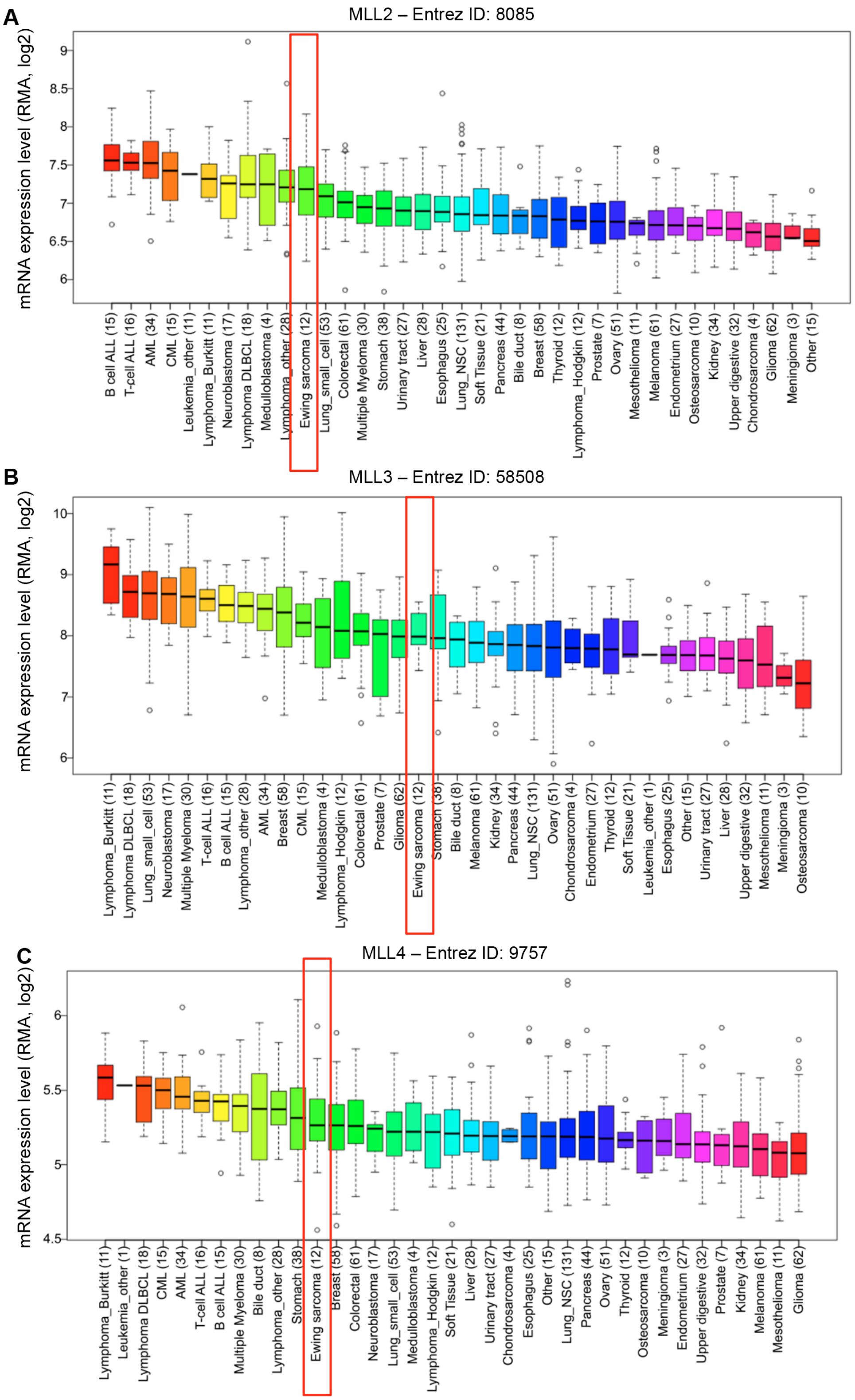
A
MLL2 – Entrez ID: 8085
mRNA expression level (RMA, log2)
9
8.5
8
7.5
7
6.5
6
B cell ALL (15)
T-cell ALL (16)
AML (34)
CML (15)
Leukemia_other (11)
Lymphoma_Burkitt (11)
Neuroblastoma (17)
Lymphoma DLBCL (18)
Medulloblastoma (4)
Lymphoma_other (28)
Ewing sarcoma (12)
Lung_small_cell (53)
Colorectal (61)
Multiple Myeloma (30)
Stomach (38)
Urinary tract (27)
Liver (28)
Esophagus (25)
Lung_NSC (131)
Soft Tissue (21)
Pancreas (44)
Bile duct (8)
Breast (58)
Thyroid (12)
Lymphoma_Hodgkin (12)
Prostate (7)
Ovary (51)
Mesothelioma (11)
Melanoma (61)
Endometrium (27)
Osteosarcoma (10)
Kidney (34)
Upper digestive (32)
Chondrosarcoma (4)
Glioma (62)
Meningioma (3)
Other (15)
B
MLL3 – Entrez ID: 58508
mRNA expression level (RMA, log2)
10
9
8
7
6
Lymphoma_Burkitt (11)
Lymphoma DLBCL (18)
Lung_small_cell (53)
Neuroblastoma (17)
Multiple Myeloma (30)
T-cell ALL (16)
B cell ALL (15)
Lymphoma_other (28)
AML (34)
Breast (58)
CML (15)
Medulloblastoma (4)
Lymphoma_Hodgkin (12)
Colorectal (61)
Prostate (7)
Glioma (62)
Ewing sarcoma (12)
Stomach (38)
Bile duct (8)
Melanoma (61)
Kidney (34)
Pancreas (44)
Lung_NSC (131)
Ovary (51)
Chondrosarcoma (4)
Endometrium (27)
Thyroid (12)
Soft Tissue (21)
Leukemia_other (1)
Esophagus (25)
Other (15)
Urinary tract (27)
Liver (28)
Upper digestive (32)
Mesothelioma (11)
Meningioma (3)
Osteosarcoma (10)
C
MLL4 – Entrez ID: 9757
mRNA expression level (RMA, log2)
6
5.5
5
4.5
Lymphoma_Burkitt (11)
Leukemia_other (1)
Lymphoma DLBCL (18)
CML (15)
AML (34)
T-cell ALL (16)
B cell ALL (15)
Multiple Myeloma (30)
Bile duct (8)
Lymphoma_other (28)
Stomach (38)
Ewing sarcoma (12)
Breast (58)
Colorectal (61)
Neuroblastoma (17)
Lung_small_cell (53)
Medulloblastoma (4)
Lymphoma_Hodgkin (12)
Soft Tissue (21)
Liver (28)
Urinary tract (27)
Chondrosarcoma (4)
Esophagus (25)
Other (15)
Lung_NSC (131)
Pancreas (44)
Ovary (51)
Thyroid (12)
Osteosarcoma (10)
Meningioma (3)
Endometrium (27)
Upper digestive (32)
Prostate (7)
Kidney (34)
Melanoma (61)
Mesothelioma (11)
Glioma (62)

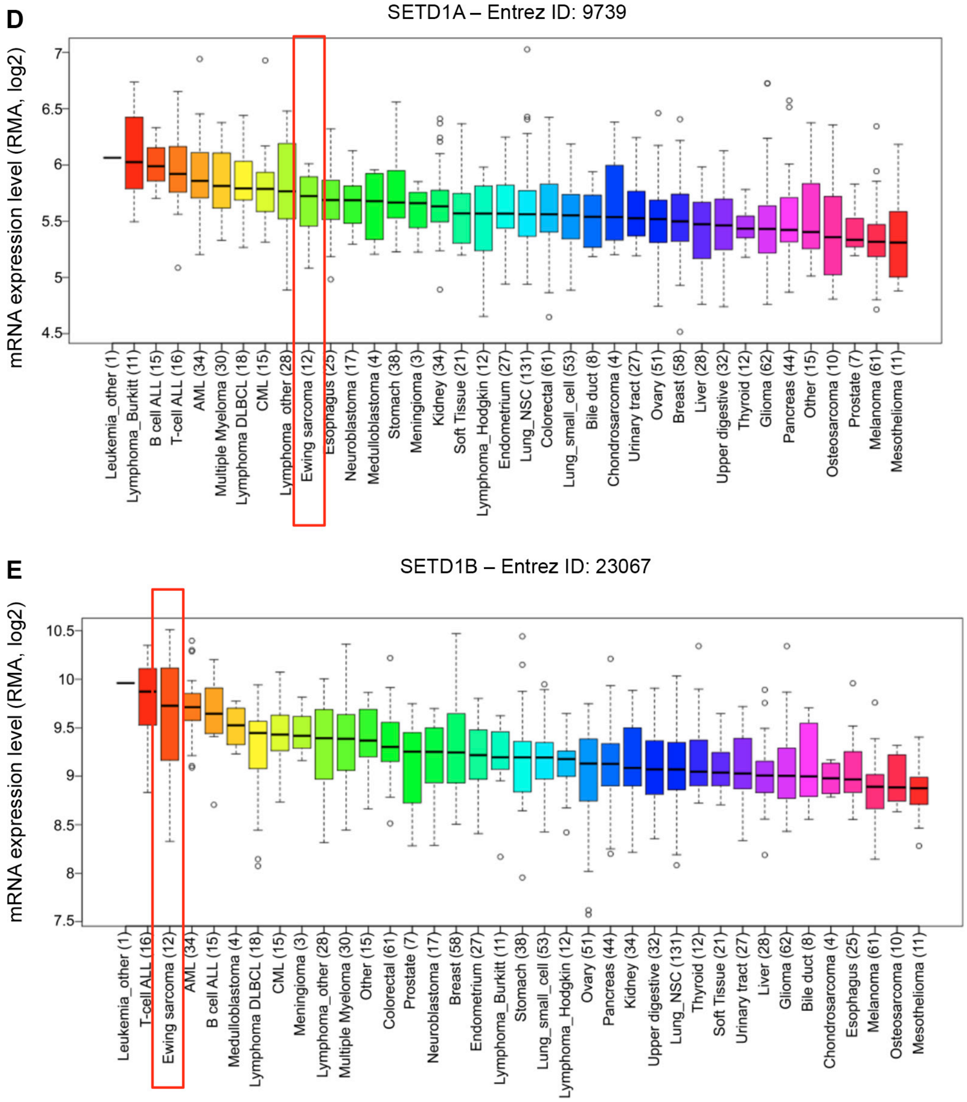

Supplementary Figure S1: MLL family histone methyltransferase expression. CCLE data depicting expression of 5 other MLL family histone methyltransferases in Ewing sarcoma cell lines compared to other cancers.

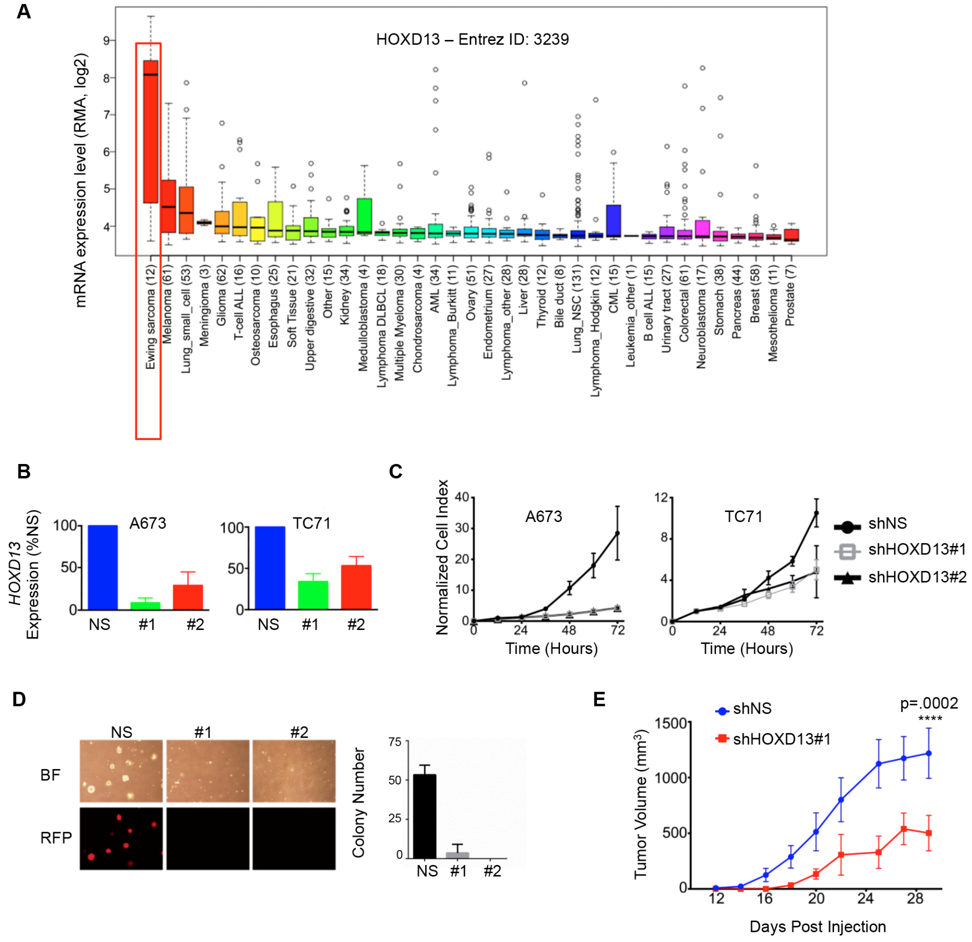

Supplementary Figure S2: HOXD13 is critical for the oncogenic phenotype of Ewing sarcoma. (**A**) Cancer Cell Line Encyclopedia data depicting high transcript expression of *HOXD13* in Ewing sarcoma relative to other cancers. (**B**) qRT-PCR data demonstrating reduced expression of *HOXD13* after 48 hr treatment with doxycycline. (**C**) xCELLigence proliferation assays showing inhibition of cell expansion after knockdown of HOXD13 with two separate constructs. (**D**) Brightfield and RFP images (left) and histogram (right) of soft agar assays in TC71 cells demonstrating that HOXD13 knockdown reduces colony formation in soft agar. (**E**) Growth curves for sub-cutaneous tumor growth in NOD-SCID mice, demonstrating a reduction in tumor growth rate with HOXD13 knockdown. $N = 6$ mice per group. ****$p = .0002$ by two-sided, unpaired t-test. Error bars in all panels depict mean ± SEM.

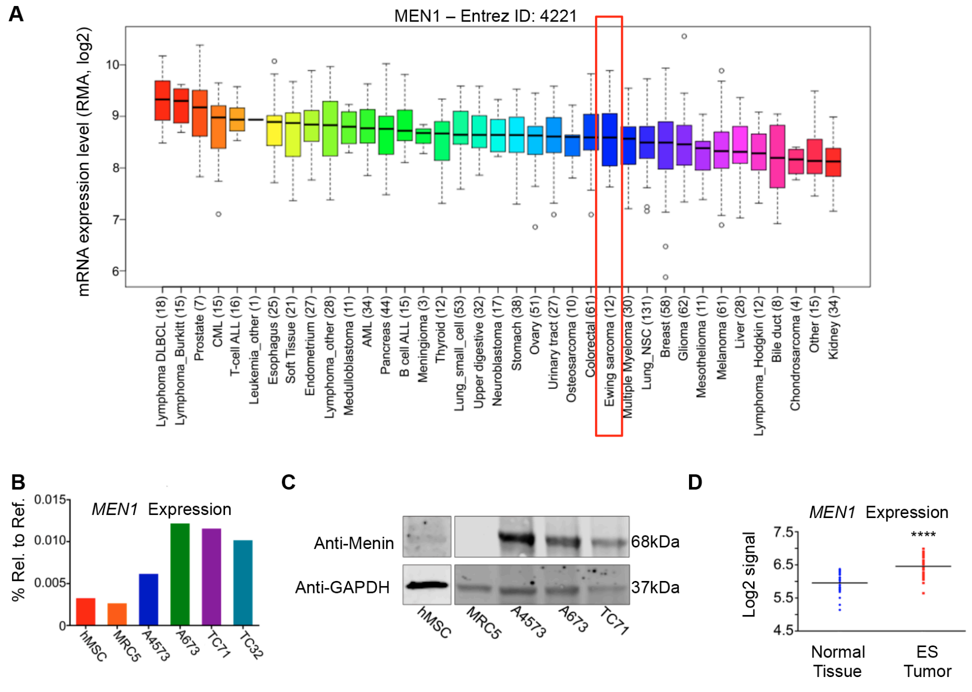

Supplementary Figure S3: Menin is expressed by Ewing sarcoma. (**A**) CCLE data showing MEN1 transcript expression. (**B**) qRT-PCR and (**C**) western blot depicting menin expression in Ewing sarcoma, MRC5 fibroblasts, and hMSC. (**D**) Expression array data for 32 Ewing sarcoma tumors compared to 11 normal adult tissues. ****$p < .0001$.

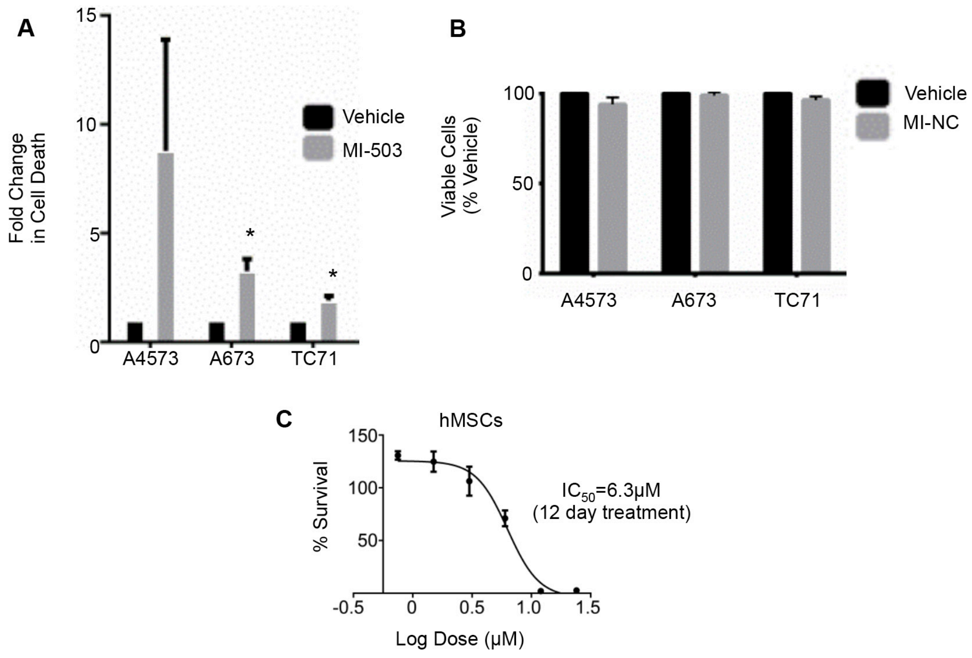

Supplementary Figure S4: (**A**) Trypan blue assay shows increased cell death after 6 day treatment with 6 µM MI-503. * denotes $p < 0.05$ by two-sided, unpaired *t*-test. (**B**) Trypan blue assay data show no difference in cell viability after 6-day treatment with 6 µM MI-NC. (**C**) Immortalized human mesenchymal stem cells (hMSCs) were treated with MI-503 for 12 days. IC_{50} curve shows that hMSCs tolerate higher doses of MI-503 for a longer time-period compared to Ewing sarcoma cells. All results are a summary of 3 independent experiments. Error bars depict mean ± SEM.

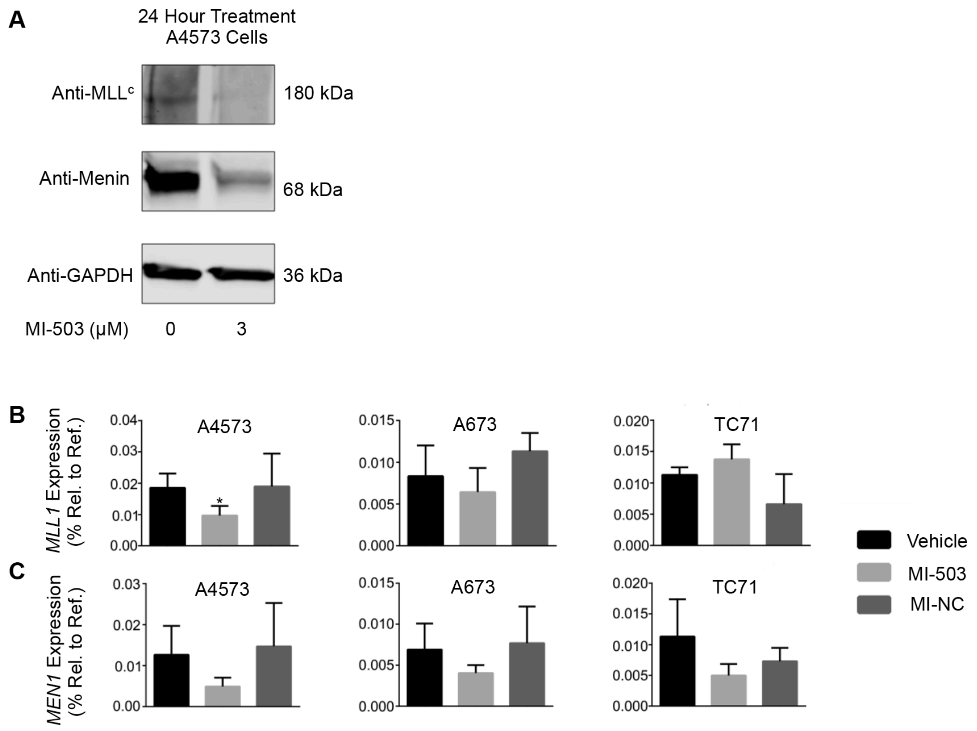

Supplementary Figure S5: (**A**) Western blot showing that down-regulation of MLL1 and menin protein expression with MI-503 treatment occurs within 24 hours. (**B**–**C**) Taqman qRT-PCR data demonstrating that 6 day treatment with 6 µM MI-503 has little consistent effect on MLL1 (B) and MEN1 (C) transcript expression compared to the same dose of MI-NC. * denotes $p < .05$ by two-sided, unpaired *t*-test.
